# Mosquito Diversity and Population Genetic Structure of Six Mosquito Species From Hainan Island

**DOI:** 10.3389/fgene.2020.602863

**Published:** 2020-10-29

**Authors:** Siping Li, Feng Jiang, Hong Lu, Xun Kang, Yanhong Wang, Zhen Zou, Dan Wen, Aihua Zheng, Chunxiang Liu, Qiyong Liu, Le Kang, Qianfeng Xia, Feng Cui

**Affiliations:** ^1^Key Laboratory of Tropical Translational Medicine of Ministry of Education and School of Tropical Medicine and Laboratory Medicine, Hainan Medical University, Haikou, China; ^2^State Key Laboratory of Integrated Management of Pest Insects and Rodents, Institute of Zoology, Chinese Academy of Sciences, Beijing, China; ^3^Beijing Institutes of Life Science, Chinese Academy of Sciences, Beijing, China; ^4^State Key Laboratory of Infectious Diseases Prevention and Control, WHO Collaborating Centre for Vector Surveillance and Management, National Institute for Communicable Disease Control and Prevention, Chinese Center for Disease Control and Prevention, Beijing, China

**Keywords:** *Aedes*, *Armigeres*, *Culex*, *Mansonia*, *Anopheles*, cytochrome c oxidase subunit I

## Abstract

Hainan is a tropical island in southern China with abundant mosquito species, putting Hainan at risk of mosquito-borne virus disease outbreaks. The population genetic diversity of most mosquito species on Hainan Island remains elusive. In this study, we report the diversity of mosquito species and the genetic diversity of the predominant species on Hainan. Field populations of adults or larvae were collected from 12 regions of Hainan Island in 2018 and 2019. A fragment of the mitochondrial cytochrome c oxidase subunit I (*coxI*) gene was sequenced from 1,228 mosquito samples and used for species identification and genetic diversity analysis. Twenty-three known mosquito species from the genera *Aedes*, *Armigeres*, *Culex*, *Mansonia*, and *Anopheles* and nine unconfirmed mosquito species were identified. *Aedes albopictus*, *Armigeres subalbatus*, and *Culex pipiens quinquefasciatus* were the most prevalent mosquito species on Hainan. The regions north of Danzhou, Tunchang, and Qionghai exhibited high mosquito diversity (26 species). The order of the total haplotype diversity and nucleotide diversity of the populations from high to low was as follows: *Culex tritaeniorhynchus*, *Ar. subalbatus*, *Culex pallidothorax*, *Culex gelidus*, *Ae. albopictus*, and *C. p. quinquefasciatus*. Tajima’s *D* and Fu’s *F*_*s*_ tests showed that *Ae. albopictus*, *C. p. quinquefasciatus*, *C. tritaeniorhynchus*, and *C. gelidus* had experienced population expansion, while the *Ar. subalbatus* and *C. pallidothorax* populations were in genetic equilibrium. Significant genetic differentiation existed in the overall populations of *Ae. albopictus*, *Ar. subalbatus*, *C. p. quinquefasciatus*, and *C. pallidothorax*. The *Ae. albopictus* populations on Hainan were characterized by frequent gene exchange with populations from Guangdong and four other tropical countries, raising the risk of viral disease outbreaks in these regions. Two subgroups were reported in the *Ar. subalbatus* populations for the first time. Our findings may have important implications for vector control on Hainan Island.

## Introduction

Hainan Island is located in southern China and has an area of 33,920 km^2^. The climate of Hainan is a tropical maritime monsoon climate with an annual average temperature of 24.2°C and an annual average rainfall of 1,684 mm. Hainan has become a China Pilot Free Trade Zone, with increasing international tourism and commercial trade, under the Belt and Road policy. The natural and cultural conditions of Hainan result in abundant mosquito species. A total of 44 mosquito species in 9 genera have been reported in Hainan based on classical morphological classification, among which 28 species have available molecular markers, such as mitochondrial cytochrome c oxidase subunit I (*coxI*) gene sequences (Zhan et al., 2000; Wang et al., 2012; Sun et al., 2014; Lian et al., 2015). The common species include *Anopheles sinensis*, *Anopheles dirus*, *Anopheles tessellatus*, *Anopheles minimus*, *Aedes albopictus*, *Aedes aegypti*, *Culex tritaeniorhynchus*, *Culex pipiens quinquefasciatus*, *Armigeres subalbatus*, etc. (Zhao et al., 2017). However, the distribution and population genetic diversity of most mosquito species in Hainan have not been reported.

The abundant mosquito diversity put Hainan at risk of mosquito-borne virus disease outbreaks. There have been several outbreaks of Japanese encephalitis virus, which is mainly transmitted by *C. tritaeniorhynchus* (Zheng et al., 2011; Zhao et al., 2017). Dengue fever, caused by *Aedes*-transmitted Dengue viruses, is endemic in Hainan (Zheng et al., 2011). Hainan has also been confirmed as a potential natural focus of other mosquito-borne viruses such as Ross River virus and chikungunya virus (Zhao et al., 2017). Therefore, knowledge of the mosquito species, distribution, and population genetic diversity on the island is key for the control of mosquitoes and mosquito-borne virus diseases on Hainan.

The *coxI* gene is a valuable and reliable diagnostic tool for studying the genetic diversity and establishing the intraspecific relationships of mosquitoes (Walton et al., 2000; Cook et al., 2005; Zhong et al., 2013). In this study, we used the *coxI* gene to investigate the diversity and population genetic diversity of field collected mosquitoes from 12 regions of Hainan in 2018 and 2019. In total, 23 known mosquito species from the genera *Aedes*, *Armigeres*, *Culex*, *Mansonia*, and *Anopheles* and nine unconfirmed mosquito species were identified. The genetic diversity of six dominant species was analyzed.

## Materials and Methods

### Mosquito Collection

Mosquitoes were collected from twelve regions: Haikou (HK), Wenchang (WC), and Lingao (LG) in the north; Sanya (SY), Lingshui (LS), and Ledong (LD) in the south; Dongfang (DF) and Danzhou (DZ) in the west; Qionghai (QH) and Wanning (WN) in the east; and Tunchang (TC) and Wuzhishan (WZS) in the central part of Hainan Island, from June to October 2019 (Supplementary Figure 1). In seven of the regions (HK, WC, SY, LS, DF, TC, and WZS) mosquitoes were collected from June to September 2018 (Supplementary Figure 1). HK, LG, WN, DZ, and LS were reported to have outbreaks of Dengue fever (Wu et al., 2007). Malaria was epidemic in WN, DF, and LD (Xiao et al., 2010). SY, LD, and HK ever outbroke with Japanese encephalitis (Fu et al., 2002; Wang J. X. et al., 2015). Each region included one sampling site, except for HK, which included three sites, and DZ, which included two sites. Several special sampling habitats included a maple deer field at TC, a virgin forest at LS, and a wetland inhabited by water birds at HK. Adult mosquitoes were captured using a human lure or light trap and stored in liquid nitrogen or RNAlater (Thermo Fisher Scientific, Waltham, MA, United States). Larvae were collected from discarded buckets and bottles, puddles, and ditches, brought to the laboratory, then raised to the adult stage before being stored in liquid nitrogen.

### DNA Extraction and Polymerase Chain Reaction Amplification

Genomic DNA was extracted from one leg of each specimen using the hot sodium hydroxide and Tris (Hot SHOT) method (Montero-Pau et al., 2008). Briefly, one leg was placed in 50 μL of alkaline lysis buffer (50 mM NaOH), followed by incubation in a thermocycler at 95°C for 30 min. Then, 6 μL of Tris-HCl (pH 7.5) was added. Appropriate forward (GGTCAACAAATCATAAAGATATTGG) and reverse (TAAACTTCAGGGTGACCAAAAAATCA) primers (Folmer et al., 1994) were used to amplify a 710 bp *coxI* gene fragment. Polymerase chain reaction (PCR) was performed in a reaction mixture containing 12.5 μL of Premix Taq (Takara Bio, Beijing, China), 1 μL of 10 mM primers, 1.5 μL of DNA, and 10 μL of distilled water. The thermal cycling conditions included a 5 min initial denaturation step at 94°C, followed by 34 cycles of 30 s of denaturation at 94°C, 30 s annealing at 55°C and 41 s elongation at 72°C, and a final elongation at 72°C for 12 min. The product was checked by 1% agarose gel electrophoresis and sent to a company (Beijing Tianyi Huiyuan Bioscience & Technology Inc., Beijing, China) for sequencing.

### Data Analysis

After removing the bases corresponding to irregular peak patterns, clean *coxI* sequences from 611 to 626 bp were obtained from 1,227 mosquito samples and deposited into the GenBank database. These sequences were aligned with the *coxI* gene sequences of different species of mosquitoes downloaded from GenBank using ClustalW of MEGA7.0 (Supplementary Table 1). When the nucleotide identity of a specimen with the homologous *coxI* sequence of a mosquito species in GenBank was over 99%, the specimen was regarded as belonging to the same species as the reference species. Sliding window analysis was performed using DnaSP V.5.10.01 to reveal the number of haplotypes, haplotype diversity, nucleotide diversity, and variable sites (Librado and Rozas, 2009). The partitioning of genetic variation within and among populations was calculated via the analysis of molecular variance (AMOVA) with 1,000 permutations implemented in Arlequin v. 3.5 (Excoffier and Lischer, 2010). The pairwise fixation index (*F*st) between the Hainan populations was calculated with the distance method. The *F*st between Hainan and other regions of China or overseas sites was calculated with haplotype frequencies. The significance level was tested with 10,000 random permutations (Slatkin and Hudson, 1991). Gene flow (Nm) was calculated as (1 – *F*st)/2*F*st (Halbert et al., 2012). Tajima’s *D* and Fu’s *Fs* values of the neutrality test were applied to examine recent population expansion when the null hypothesis of neutrality was rejected due to significant negative values (Tajima, 1989; Fu, 1997). Phylogenetic trees for the *Aedes*, *Anopheles*, and *Culex* genera and *Ar. subalbatus* were constructed based on the nucleotide sequences of *coxI* using the neighbor-joining method (p-distance model and pairwise deletion) in MEGA 7.0. The statistical significance of tree branching was tested by performing 1,000 bootstrap replications.

## Results

### Mosquito Species Identification

**FIGURE 1:**
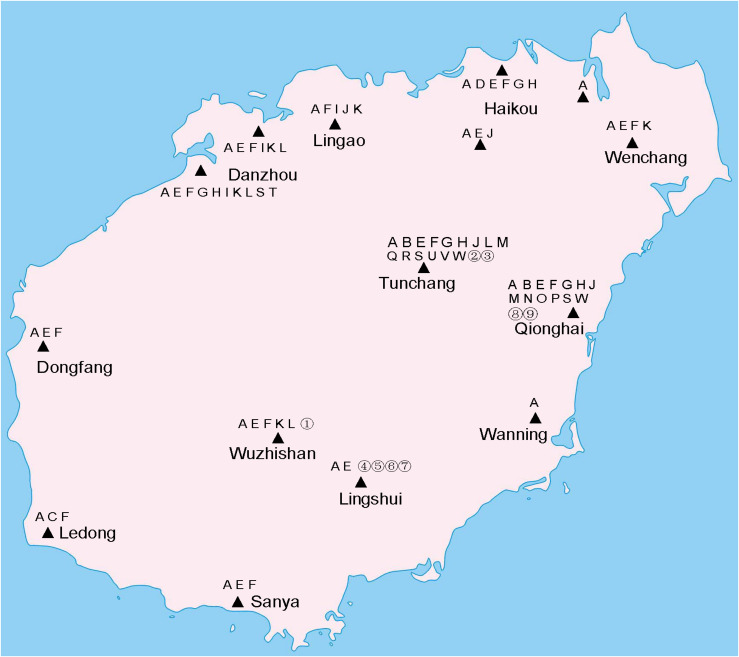
Distribution of mosquito species on Hainan. Triangles indicate collection sites. A, *Ae. albopictus*. B, *Ae. vexans*. C, *Ae. aegypti*. D, *Ae. malayensis*. E, *Ar. subalbatus*. F, *C. p. quinquefasciatus*. G, *C. gelidus*. H, *C. tritaeniorhynchus*. I, *C. sitiens*. J, *C. vishnui*. K, *C. pallidothorax*. L, *C. fuscanus*. M, *C. fuscocephala*. N, *C. pseudovishnui*. O, *C. cinctellus*. P, *C. bitaeniorhynchus*. Q, *An. barbirostris*. R, *An. tessellatus*. S, *An. vagus*. T, *An. sinensis*. U, *An. aconitus*. V, *An. kochi*. W, *M. uniformis*. Unconfirmed mosquito species are numbered from 1 to 9.

The *coxI* sequences of single mosquitoes from Hainan were obtained and aligned with the *coxI* sequences of different species of mosquitoes downloaded from GenBank. Twenty-three known mosquito species in five genera were identified in the two years of the investigation in Hainan (Table 1), including *Ae. albopictus*, *Ae. aegypti*, *Aedes vexans*, *Aedes malayensis*, *Ar. subalbatus*, *C. p. quinquefasciatus*, *Culex gelidus*, *Culex vishnui*, *C. tritaeniorhynchus*, *Culex pallidothorax*, *Culex fuscanus*, *Culex fuscocephala*, *Culex pseudovishnui*, *Culex sitiens*, *Culex cinctellus*, *Culex bitaeniorhynchus*, *Mansonia uniformis*, *Anopheles barbirostris*, *An. tessellatus*, *Anopheles vagus*, *An. sinensis*, *Anopheles kochi*, and *Anopheles aconitus*. In addition to the known species, nine samples showed a nucleotide identity between 87 and 96% with known mosquito species. Therefore, they were treated as unconfirmed mosquito species and excluded from any further analyses (Supplementary Table 2). *Ae. albopictus* was the most prevalent mosquito species on Hainan Island and was found at all 12 sampling locations. *C. p. quinquefasciatus* and *Ar. subalbatus* were second to *Ae. albopictus* in terms of their distribution across the island. From the overall distribution of the mosquitoes, it was clear that the regions north of the line from Danzhou to Tunchang and Qionghai were characterized by plentiful mosquito species; 22 known and 4 unconfirmed mosquito species were found in these areas. In contrast, only 6 known and 5 unconfirmed mosquito species were found in regions south of this line (Figure 1).

**TABLE 1:** Species and numbers of mosquitoes collected in Hainan based on the nucleotide sequences of *coxI*.

Species	Location											
	
	HK	WC	LD	SY	LG	DZ	DF	QH	WN	LS	TC	WZS
*Aedes albopictus*	28	23	35	41	12	21	33	15	21	48	50	25
*Ae. vexans*								4			2	
*Ae. aegypti*			3									
*Ae. malayensis*	3											
*Armigeres subalbatus*	26	24		15		33	3	1		15	5	29
*Culex pipiens quinquefasciatus*	78	2	109	65	1	135	38	16			2	93
*C. gelidus*	15					7		7			51	
*C. vishnui*	4				2			4			17	
*C. pseudovishnui*								4				
*C. tritaeniorhynchus*	3					6		45			37	
*C. pallidothorax*		12			1	22						65
*C. fuscanus*						16					1	9
*C. sitiens*					98	13						
*C. cinctellus*								1				
*C. bitaeniorhynchus*								1				
*C. fuscocephala*								19			5	
*Mansonia uniformis*								1			35	
*Anopheles barbirostris*											4	
*An. tessellatus*											4	
*An. aconitus*								1			1	
*An. vagus*						2		2			2	
*An. sinensis*						1						
*An. kochi*											1	

**FIGURE 2:**
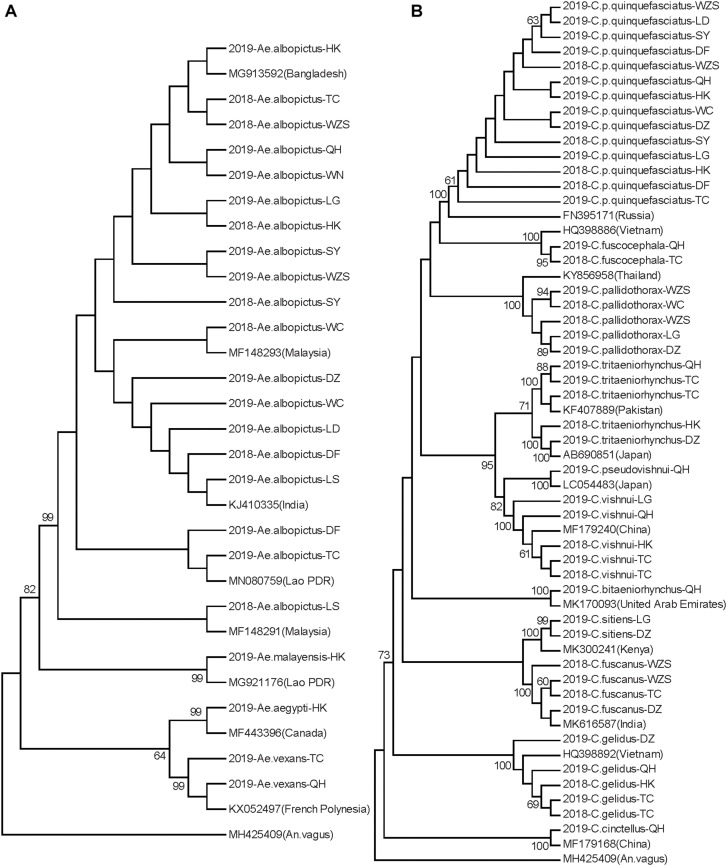
Neighbor-joining phylogenetic trees of *Aedes* and *Culex* genus mosquitoes from Hainan based on the nucleotide sequences of *coxI*. **(A)** *Aedes* genus. **(B)** *Culex* genus. The Hainan samples are named according to the year-species-collection site. Sequences of mosquitoes with accession numbers in GenBank from other regions (in brackets) are referenced. The sequence of *Anopheles vagus* is used as the outgroup. Bootstrap values over 60 are indicated.

**FIGURE 3:**
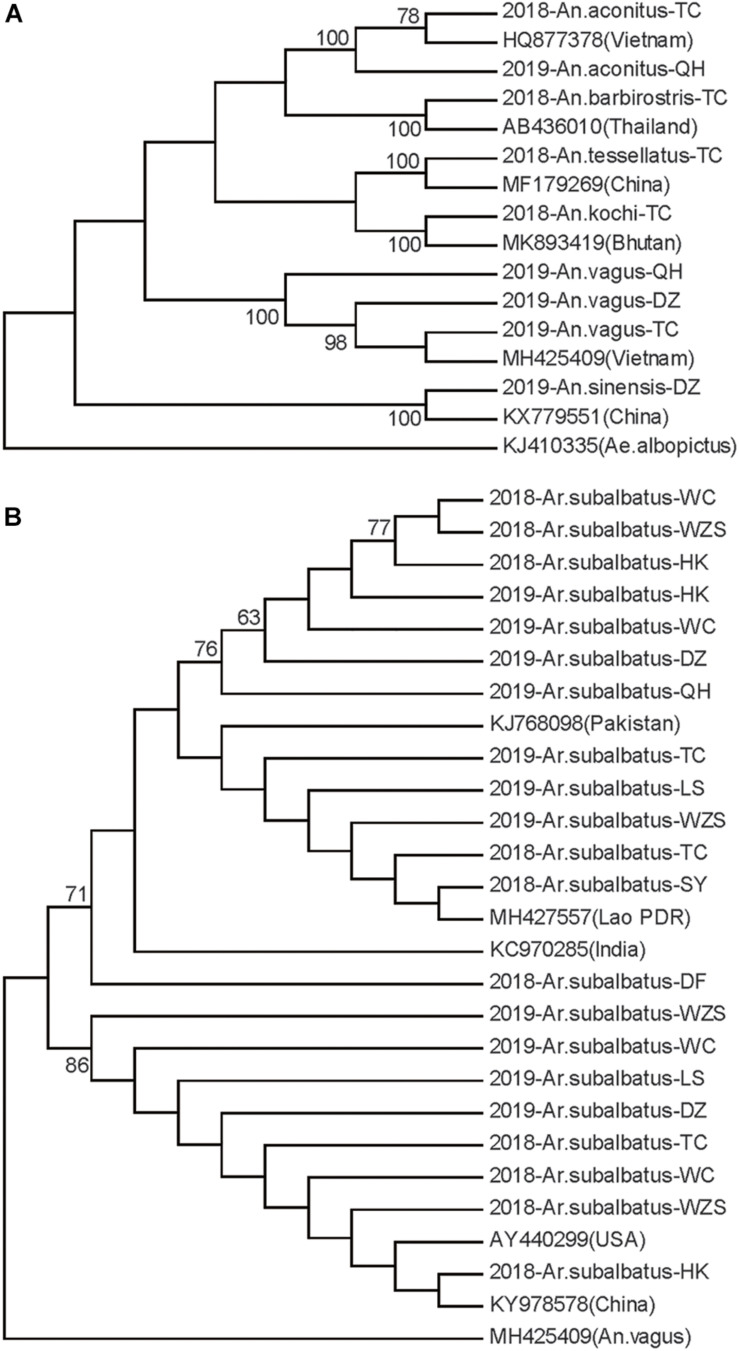
Neighbor-joining phylogenetic trees of the *Anopheles* genus and *Armigeres subalbatus* from Hainan based on the nucleotide sequences of *coxI*. **(A)** *Anopheles* genus. **(B)** *Ar. subalbatus*. The Hainan samples are named according to the year-species-collection site. Sequences of mosquitoes with accession numbers in GenBank from other regions (in brackets) are referenced. The sequence of *Aedes albopictus* or *Anopheles vagus* is used as an outgroup. Bootstrap values over 60 are indicated.

Neighbor-joining phylogenetic trees were constructed for the Hainan mosquitoes of the *Aedes*, *Culex*, and *Anopheles* genera with reference sequences of mosquitoes from other regions. In the *Aedes* genus, the four species formed distinct clades with 99% bootstrap values (Figure 2A). In the *Culex* genus, each species formed a distinct clade supported by a 100% bootstrap value except for *C. tritaeniorhynchus*, which was split into two subclades with a 71% bootstrap value. Furthermore, *C. vishnui*, *C. pseudovishnui*, and *C. tritaeniorhynchus* clustered together with a 95% bootstrap value, forming the acknowledged *C. vishnui* complex (Figure 2B; Kumar et al., 2017). In the *Anopheles* genus, the six species formed distinct clades with 100% bootstrap values (Figure 3A).

### Genetic Diversity of *Ae. albopictus* Populations

*Ae. albopictus* was collected in 12 regions of Hainan. In total, 33 variable sites and 46 haplotypes were detected (Table 2). Haplotypes 1 and 5 were the most widely distributed haplotypes (Supplementary Table 3). The total haplotype diversity (*H*_*d*_) was 0.62, and the nucleotide diversity (π) was 0.17. The highest diversity was found in the DF population, and the lowest diversity was found in the WN population (Table 2). Pairwise population differentiation was evaluated with the fixation index (*F*st) using the distance method (Table 3). High significant pairwise population differentiation was observed between DF and the other populations (*F*st between 0.17 and 0.24). However, the Nm values between DF and the other populations were larger than 1 (from 1.55 to 2.44), indicating frequent gene exchange between them (Table 3). The DZ and HK populations showed significant but low differentiation (*F*st < 0.1) from some populations due to more frequent gene exchange. The molecular variance analysis (AMOVA) showed that the majority of the genetic variance occurred within populations (92.68%) (Supplementary Table 4). The total *F*st was 0.07 (*P* < 0.001), and Nm was 6.64, reflecting low population differentiation. Tajima’s *D* value (−2.10) and Fu’s *F*_*s*_ value (−29.15) for the overall populations both reached a significant level, reflecting significant population expansion (Table 2). Regarding the specific populations, SY and TC presented significant negative *D* and *F*_*s*_ values. WC and DZ exhibited significant negative *F*_*s*_ values.

**TABLE 2:** Haplotype and nucleotide diversity of the *coxI* gene of six mosquito species and the neutrality test.

	Location	N	H	Variable sites	Haplotype diversity (*H*_*d*_ ± SD)	Nucleotide diversity (π × 10^2^)	Tajima’s *D*	Fu’s *F*_*S*_
*Aedes albopictus*	HK	28	6	6	0.50 ± 0.11	0.20	−0.62	−1.17
	WC	23	8	7	0.75 ± 0.08	0.21	−1.07	−3.62*
	LD	35	5	4	0.63 ± 0.06	0.14	−0.37	−0.97
	SY	41	9	8	0.62 ± 0.07	0.14	−1.54*	−5.37*
	LG	12	2	1	0.41 ± 0.13	0.07	0.54	0.74
	DZ	21	9	8	0.73 ± 0.10	0.26	−0.95	−4.16*
	DF	33	8	9	0.77 ± 0.04	0.26	−0.82	−1.86
	QH	15	4	4	0.47 ± 0.15	0.13	−1.07	−0.77
	WN	21	2	1	0.38 ± 0.10	0.06	0.66	0.94
	LS	48	6	6	0.54 ± 0.06	0.11	−1.30	−2.33
	TC	50	14	12	0.68 ± 0.07	0.18	−1.75*	−10.95**
	WZS	25	4	4	0.41 ± 0.11	0.10	−1.12	−0.88
	Total	352	46	33	0.62 ± 0.03	0.17	−2.10*	−29.15*
*Armigeres subalbatus*	HK	26	4	9	0.40 ± 0.11	0.33	−0.45	2.26
	WC	24	6	10	0.82 ± 0.05	0.53	0.70	1.55
	SY	15	4	7	0.62 ± 0.12	0.22	−1.31	0.38
	DZ	33	6	11	0.58 ± 0.09	0.33	−0.82	0.55
	LS	15	5	9	0.63 ± 0.13	0.26	−1.56*	−0.33
	WZS	29	7	12	0.78 ± 0.06	0.53	0.26	1.08
	Total	142	16	22	0.74 ± 0.03	0.42	−0.96	−2.71
*Culex pipiens quinquefasciatus*	HK	78	2	1	0.36 ± 0.05	0.06	0.93	1.48
	LD	54	9	20	0.57 ± 0.07	0.22	−2.15*	−6.03*
	SY	65	2	1	0.12 ± 0.05	0.02	−0.56	−0.32
	DZ	113	10	44	0.51 ± 0.04	0.21	−2.63**	−3.13
	DF	38	3	2	0.10 ± 0.07	0.02	−1.49*	−1.41*
	QH	16	2	1	0.13 ± 0.11	0.02	−1.16	−0.70
	WZS	93	5	9	0.08 ± 0.04	0.03	−2.25**	−3.72*
	Total	457	23	62	0.35 ± 0.03	0.11	−2.63**	−29.07**
*Culex tritaeniorhynchus*	DZ	6	5	32	0.93 ± 0.12	1.94	−0.91	1.12
	QH	45	37	56	0.99 ± 0.01	0.96	−1.89*	−25.29*
	TC	37	25	36	0.97 ± 0.02	0.67	−1.83*	−17.90*
	Total	88	63	70	0.98 ± 0.01	0.91	−1.96*	−25.32*
*Culex gelidus*	HK	15	5	4	0.71 ± 0.09	0.14	−0.92	−1.86*
	DZ	7	3	2	0.67 ± 0.09	0.14	0.21	−0.24
	QH	7	4	3	0.81 ± 0.13	0.18	−0.30	−1.22
	TC	51	14	16	0.79 ± 0.05	0.27	−1.69*	−7.45*
	Total	80	15	17	0.75 ± 0.04	0.22	−1.76*	−8.55**
*Culex pallidothorax*	WC	12	4	5	0.76 ± 0.08	0.30	0.50	0.78
	DZ	22	7	7	0.86 ± 0.03	0.30	1.07	−0.26
	WZS	65	9	9	0.63 ± 0.06	0.29	−0.12	−1.12
	Total	99	13	13	0.79 ± 0.03	0.38	−0.13	−1.97

*P < 0.05, **P < 0.01. N, sample number; H, haplotype number.

**TABLE 3:** Pairwise genetic differentiation (*F*st; lower triangle) and gene flow (Nm; upper triangle) between *Aedes albopictus* populations on Hainan.

Location	HK	WC	LD	SY	LG	DZ	DF	QH	WN	LS	TC	WZS
HK		11.09	4.30	8.34	58.75	6.01	1.87	32.22	14.55	8.63	14.85	20.74
WC	0.04		12.94	31.45	–	11.30	2.31	–	78.28	–	–	53.39
LD	0.10**	0.04		21.23	56.63	6.05	2.44	28.86	12.72	17.82	54.03	12.72
SY	0.06*	0.02	0.02		–	9.10	1.71	–	–	–	–	–
LG	0.01	−0.02	0.01	−0.04		148.00	2.00	–	–	–	–	–
DZ	0.08**	0.04	0.08*	0.05*	0.01		1.80	20.47	14.54	8.31	10.28	11.88
DF	0.21**	0.18**	0.17**	0.23**	0.20**	0.22**		2.08	1.55	1.57	1.96	1.71
QH	0.05	−0.01	0.02	−0.02	−0.06	0.02	0.19**		–	–	–	–
WN	0.03	0.01	0.04	−0.02	−0.07	0.03	0.24**	−0.03		–	–	–
LS	0.05*	−0.01	0.03	−0.01	−0.05	0.06*	0.24**	−0.02	−0.03		–	–
TC	0.03*	−0.01	0.01	−0.01	−0.04	0.05*	0.20**	−0.03	−0.02	−0.01		–
WZS	0.02	0.01	0.04	−0.01	−0.06	0.04	0.22**	−0.03	−0.04	−0.02	−0.01	

*P < 0.05, **P < 0.01. When *F*st is negative, Nm is not available.

The *F*st values between Hainan populations and those of other regions from China or overseas were also calculated according to the haplotype frequencies (Table 4). The haplotype sequences of the *Ae. albopictus* populations from Henan, Fujian, Yunnan, Guangdong in China, the Congo, the United States of America (United States), Italy, the Lao People’s Democratic Republic, Singapore, Japan, Thailand, and Pakistan were downloaded from GenBank. The Hainan populations exhibited the largest divergence from the United States population (*F*st = 0.42, *P* < 0.01) and the least divergence from the Congo population (*F*st = 0.09, *P* < 0.01). There was frequent gene flow between the Hainan populations and the Henan, Yunnan, Guangdong, Congo, Lao People’s Democratic Republic, Singapore, or Thailand populations (Nm from 1 to 5.3).

**TABLE 4:** Pairwise genetic differentiation (*F*st; lower triangle) and gene flow (Nm; upper triangle) between different geographical populations of *Aedes albopictus*.

Location	HN	HEN	FJ	YN	GD	CG	United States	IT	LA	SG	JP	TL	PT
HN		1.02	0.89	2.97	1.74	5.30	0.68	0.89	3.00	1.00	0.86	1.67	0.88
HEN	0.33**		2.21	2.36	4.42	0.25	3.17	1.68	1.67	1.23	3.77	1.88	0.89
FJ	0.36**	0.18*		1.47	2.92	0.26	1.30	1.12	0.99	0.99	1.61	1.36	1.75
YN	0.14*	0.17**	0.25**		17.94	0.43	0.76	1.63	10.76	1.94	1.49	9.31	1.61
GD	0.22**	0.10**	0.15**	0.03*		0.36	1.57	2.30	2.15	2.39	2.36	5.49	1.82
CG	0.09**	0.67**	0.65**	0.58*			0.21	0.24	0.81	0.32	0.23	0.40	0.22
United States	0.42**	0.14	0.28**	0.30**	0.71*			0.85	0.73	0.66	2.26	0.82	0.45
IT	0.36**	0.23*	0.31**	0.18**	0.68**	0.37**			1.00	1.00	1.23	1.46	0.74
LA	0.14**	0.30**	0.34**	0.19	0.38**	0.41**	0.33**			1.14	0.97	3.92	1.08
SG	0.33**	0.29**	0.34**	0.17**	0.61**	0.43**	0.33**	0.31**			2.37	1.66	0.92
JP	0.37**	0.12	0.24**	0.18**	0.68**	0.18**	0.29**	0.34**	0.17**			1.38	0.7
TL	0.23**	0.21**	0.27**	0.05	0.55	0.38**	0.25**	0.11**	0.23**	0.27**			1.49
PT	0.36**	0.36**	0.40**	0.24**	0.69**	0.53**	0.40**	0.32**	0.35**	0.42**	0.25**		

HN, Hainan, 352 samples. HEN, Henan, 10. FJ, Fujian, 28. YN, Yunnan, 9. GD, Guangdong, 11. CG, Congo, 127. United States, 35. IT, Italy, 14. LA, Lao DPR, 154. SG, Singapore, 36. JP, Japan, 15. TL, Thailand, 29. PT, Pakistan, 11. *P < 0.05, **P < 0.01. When *F*st is negative, Nm is not available.

### Genetic Diversity of *Ar. subalbatus* Populations

*Ar. subalbatus* was collected from nine regions of Hainan. The phylogenetic analysis showed that two subgroups were clustered with over 70% bootstrap values. The TC, WZS, LS, HK, DZ, and WC populations contained individuals from both subgroups (Figure 3B). Genetic diversity was analyzed among the HK, WC, SY, DZ, LS, and WZS populations because they presented a sample size of more than 10 individuals.

There were 16 haplotypes with 22 variable sites, and only one haplotype appeared in all populations (Table 2 and Supplementary Table 5). The two subgroups did not share any haplotypes. The total haplotype diversity and nucleotide diversity were 0.74 (*H*_*d*_) and 0.42 (π), respectively. HK exhibited the lowest haplotype diversity, and SY exhibited the lowest nucleotide diversity. WC showed the highest haplotype and nucleotide diversity (Table 2). Tajima’s *D* tests and Fu’s *F*_*s*_ test for the overall populations did not present statistically significant negative values, suggesting that the *Ar. subalbatus* populations of Hainan were in genetic equilibrium. Only LS exhibited a significant negative *D* value, implying population expansion (Table 2). Significant genetic differentiation existed in half of the pairs of the six populations, especially those including the SY population, which showed significant differentiation from all other populations except for LS (*F*st between 0.10 and 0.26, Table 5), largely because all individuals from SY belonged to a single subgroup (Figure 3B). Genetic exchange frequently occurred between most populations (Nm values from 1.41 to 88.97) (Table 5). The total *F*st was 0.10 (*P* < 0.01), and Nm was 4.50. Most of the total variation existed within populations (89.52%) (Supplementary Table 6). However, when AMOVA was applied to the two subgroups, 77.06% of the total variation was found to exist between the subgroups (Supplementary Table 6). The *F*st between subgroups was 0.77 (*P* < 0.01), and Nm was less than 1, indicating that gene flow failed to prevent the subgroup differentiation caused by genetic drift.

**TABLE 5:** Pairwise genetic differentiation (*F*st; lower triangle) and gene flow (Nm; upper triangle) between *Armigeres subalbatus* populations on Hainan.

Location	HK	WC	SY	DZ	LS	WZS
HK		14.68	1.41	–	2.27	1.86
WC	0.03		4.38	17.68	30.54	10.68
SY	0.26**	0.10*		2.25	88.97	4.30
DZ	−0.05	0.03	0.18**		4.12	2.22
LS	0.18**	0.02	0.01	0.11**		19.71
WZS	0.21**	0.04	0.10*	0.18**	0.02	

*P < 0.05, **P < 0.01. When *F*st is negative, Nm is not available.

The genetic differentiation of the Hainan *Ar. subalbatus* populations from those of Pakistan or India was analyzed using haplotype frequencies. The Hainan populations exhibited greater differentiation from the Pakistan population (*F*st = 0.30, *P* < 0.01) than the Indian population (*F*st = 0.13, *P* < 0.05) but showed frequent gene flow with both populations (Supplementary Table 7).

### Genetic Diversity of *C. p. quinquefasciatus* Populations

The collection of *C. p. quinquefasciatus* populations was performed in seven regions. In total, 23 haplotypes with 62 variable sites were identified, and only one haplotype appeared in all populations (Table 2 and Supplementary Table 8). The total haplotype diversity and nucleotide diversity were comparatively low (*H*_*d*_ = 0.35, π = 0.11), but LD and DZ showed the highest diversity (*H*_*d*_ of approximately 0.5, π of approximately 0.2). Tajima’s *D* tests and Fu’s *F*_*s*_ test for the overall populations presented statistically significant negative values (Table 2), indicating significant population expansion. LD, DF and WZS exhibited significant negative *D* and *F*_*s*_ values, and DZ showed a significant negative *D*. The largest pairwise population differentiation existed between HK and SY (*F*st = 0.18, *P* < 0.01). HK exhibited significant differentiation from all six other populations, followed by LD and DZ, which showed significant differentiation from five populations (Table 6). Frequent gene flow occurred between most populations (Nm from 2.31 to 143.25) (Table 6). The total *F*st was 0.10 (*P* < 0.01), and Nm was 4.50. The majority of the variation (90.43%) existed within populations (Supplementary Table 9). Compared with the populations from Turkey, the United Kingdom, Serbia, and Canada, the Hainan populations exhibited no significant genetic differentiation. The pairwise divergence between all populations was not significant (Supplementary Table 10).

**TABLE 6:** Pairwise genetic differentiation (*F*st; lower triangle) and gene flow (Nm; upper triangle) between *Culex pipiens quinquefasciatus* populations on Hainan.

Location	HK	LD	SY	DZ	DF	QH	WZS
HK		3.36	2.31	3.58	2.80	3.55	2.63
LD	0.13**		10.15	4.61	7.87	17.68	5.33
SY	0.18**	0.05**		5.62	19.54	19.03	57.87
DZ	0.12**	0.10**	0.08**		7.49	12.62	5.22
DF	0.15**	0.06**	0.02	0.06**		143.25	–
QH	0.12*	0.03	0.03	0.04	0.01		–
WZS	0.16**	0.09**	0.01	0.09**	−0.01	−0.01	

*P < 0.05, **P < 0.01. When *F*st is negative, Nm is not available.

### Genetic Diversity of Populations of Other Mosquito Species

*C. tritaeniorhynchus* was collected at TC, QH, and DZ. There were 63 haplotypes and 70 variable sites detected in these populations in total (Table 2 and Supplementary Table 11). The total haplotype diversity and nucleotide diversity were quite high (*H*_*d*_ = 0.98, π = 0.91), and these populations experienced expansion during their history with significant negative Tajima’s *D* and Fu’s *F*_*s*_ values (Table 2). No significant genetic differentiation was observed for these populations (Supplementary Table 12).

*C. gelidus* was collected at TC, HK, DZ, and QH. Fifteen haplotypes and 17 variable sites were detected (Table 2 and Supplementary Table 13). The total haplotype diversity was 0.75, and the total nucleotide diversity was 0.22. The Tajima’s *D* and Fu’s *F*_*s*_ values for the overall populations presented significant negative values, indicating population expansion (Table 2). No significant genetic differentiation was observed for these populations (Supplementary Table 14).

*C. pallidothorax* was collected at WC, DZ and WZS. Thirteen haplotypes and 13 variable sites were found in these populations (Table 2 and Supplementary Table 15). The total haplotype diversity was 0.79, and the total nucleotide diversity was 0.38. The overall populations were in genetic equilibrium due to non-significant negative Tajima’s *D* and Fu’s *F*_*s*_ values (Table 2). Significant genetic differentiation existed between the pairs of the three populations, and the largest differentiation appeared between DZ and WZS (*F*st = 0.36, *P* < 0.01) due to limited genetic exchange between them (Nm less than 1) (Table 7). The total *F*st was 0.33 (*P* < 0.001), and Nm was 1.01. A considerable proportion (33.06%) of genetic variance existed among populations (Supplementary Table 16).

**TABLE 7:** Pairwise genetic differentiation (*F*st; lower triangle) and gene flow (Nm; upper triangle) between *Culex pallidothorax* populations on Hainan.

Location	WC	DZ	WZS
WC		2.12	2.70
DZ	0.19**		0.90
WZS	0.16*	0.36**	

*P < 0.05, **P < 0.01. When *F*st is negative, Nm is not available.

## Discussion

In the investigation of mosquito populations at Hainan from 2018 to 2019, we found 23 known species in five genera, including four *Aedes*, 11 *Culex*, six *Anopheles*, one *Mansonia*, and one *Armigeres* species based on the *coxI* sequences. In addition, nine specimens were not confirmed due to their low identities with the *coxI* sequences of known mosquitoes in GenBank. Although *coxI* has emerged as the most commonly used marker for barcoding, this marker sometimes does not contain enough information to distinguish certain mosquito species of *Anopheles and Culex* (Bourke et al., 2013; Laurito et al., 2013). Another limitation of the barcoding approach is the recombination within mitochondrial genes may lead to complex sequence patterns when species with divergent mitochondrial DNA genomes interbreed (Chan et al., 2014). To avoid potential errors from the unique *coxI* barcoding, we used an over 99% cutoff in the nucleotide identity with the published homologous reference sequences of mosquito species.

The predominant species at Hainan probably changed with time. In previous studies, *Ae. albopictus*, *Ae. aegypti*, *C. tritaeniorhynchus*, *C. p. quinquefasciatus*, *An. dirus*, *An. sinensis*, *An. tessellates*, *An. minimus*, and *An. barbirostris* have been found to be broadly distributed on Hainan (Zhao et al., 2017). However, in this study, *Ae. albopictus*, *C. p. quinquefasciatus*, and *Ar. subalbatus* were the most prevalent species. *Ar. subalbatus* was detected in nine regions, whereas it was only found in Haikou, Sanya and Baoting before 2014 (Su et al., 1994; Zhan et al., 2000; Wang X. et al., 2015). *Ae. aegypti*, *An. sinensis*, *An. tessellates*, and *An. barbirostris* were found only in one region. *C. tritaeniorhynchus* was collected in four regions. We did not collect any *An. dirus* or *An. minimus* specimens.

The richness and dominant mosquito species of Hainan Island are different from those of other tropical islands. In an investigation conducted from 2005 to 2012 on Taiwan Island, 26 mosquito species from 8 genera were identified (Su et al., 2014). The most prevalent species on Taiwan Island were *C. tritaeniorhynchus*, *C. sitiens* and *An. sinensis*, differing considerably from the situation on Hainan. Thirteen species were commonly observed on Hainan and Taiwan Islands. Eight species of *Aedes* and *Culex* were found in 2013 on Tongatapu Island, which is located in the South Pacific Ocean (Swan and Harding, 2017). *Ae. aegypti* was the most prevalent species on Tongatapu Island, followed by *Ae. albopictus*. *C. sitiens*, *C. p. quinquefasciatus*, *Ae. albopictus*, *Ae. aegypti*, and *Ae. vexans*, which existed on all three islands.

The Hainan *Ae. albopictus* populations showed frequent gene flow with the Yunnan and Guangdong populations but not with the Fujian populations. Fang et al. (2018) reported that Hainan *Ae. albopictus* only exhibited frequent gene flow with the Yunnan population and that gene exchange between Hainan and Guangdong or Fujian populations was blocked. We also found that the Hainan *Ae. albopictus* populations showed frequent gene flow with the Congo, Lao People’s Democratic Republic, Singapore, and Thailand populations. Our results remind us that the risk of outbreaks of *Ae. albopictus*-borne human viruses, such as Dengue virus and Zika virus, is elevated in these tropical areas considering the frequent gene flow between them, especially between Guangdong and Hainan.

*Ar. subalbatus* is known to be the vector for parasites of many human diseases, such as Japanese encephalitis virus and the filarial worm *Wuchereria bancrofti* (Das et al., 1983). Two subgroups are reported in the *Ar. subalbatus* populations on Hainan for the first time. The *Ar. subalbatus* specimens registered in GenBank from Pakistan, India and the Lao People’s Democratic Republic all belong to one subgroup, while the *Ar. subalbatus* specimens from a lab in United States (AY440299) (Bartholomay et al., 2004) and Yunnan in China (KY978578) (Li and Chen, 2018) are closely related to the other subgroup. *Ar. subalbatus* has become one of the most prevalent species at Hainan according to this investigation. The overall populations remained in genetic equilibrium. Six populations contained individuals from the two subgroups. A high ratio of variation (77.06%) existed between the two subgroups. This is a dominant cause of the high haplotype and nucleotide diversity of the *Ar. subalbatus* populations on Hainan. The genetic divergence between the two subgroups was quite high (*F*st = 0.77), and gene flow between them was blocked. It is possible that in the future, the accumulation of genetic differences will lead to reproductive isolation between the two subgroups and, thus, the formation of new species.

In conclusion, our results showed a high diversity of mosquito species and their population genetic characteristics on Hainan Island. These results may have important implications for vector control and shed light on understanding the evolutionary processes of these mosquito species.

## Data Availability Statement

The datasets presented in this study can be found in online repositories. The names of the repository/repositories and accession number(s) can be found below: https://www.ncbi.nlm.nih.gov/genbank/, MT541015–MT541156, MT541161–MT541779, MT566458–MT566913, MT575769–MT575771, MT576036, MT586701, MT590372, MT596915, MT606009, and MT613992.

## Author Contributions

FC, QX, and LK designed the experiments. FC and SL wrote the manuscript. SL performed the experiments and conducted the data analysis. SL, FJ, HL, XK, YW, ZZ, DW, AZ, CL, QL, and FC collected mosquitoes from the fields. All authors contributed to the article and approved the submitted version.

## Conflict of Interest

The authors declare that the research was conducted in the absence of any commercial or financial relationships that could be construed as a potential conflict of interest.
