# Supplementary material for: Mosquito Diversity and Population Genetic Structure of Six Mosquito Species From Hainan Island
Source: Front Genet. 2020 Oct 29;11:602863. doi: 10.3389/fgene.2020.602863 (PMC7658394; doi:10.3389/fgene.2020.602863)
Supplement: Supplementary file 1 [file Data_Sheet_1.PDF]

Supplementary Material

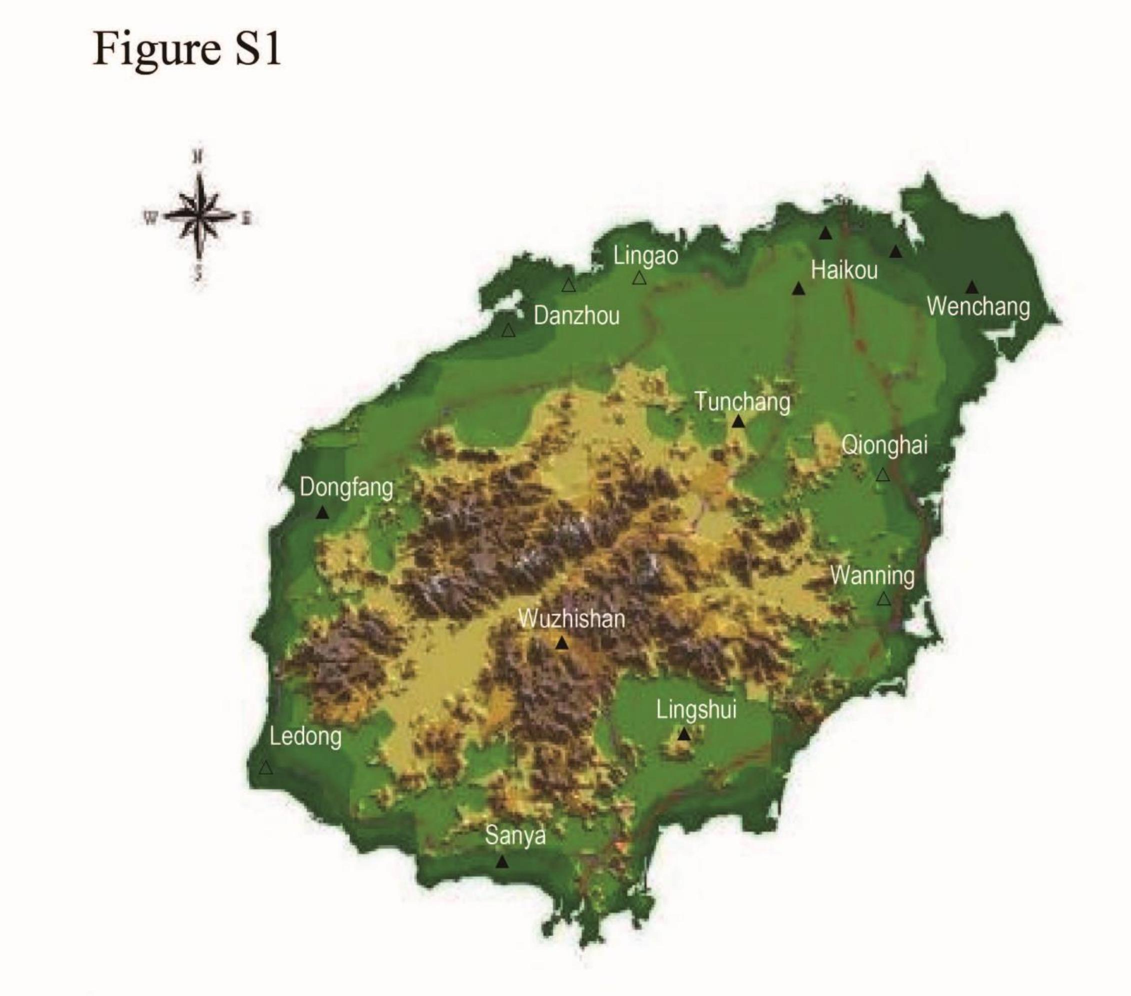

Supplementary Figure 1. **Mosquito collection sites on Hainan.** Black triangles indicate the collection sites in both 2018 and 2019. White triangles indicate the collection sites only in 2019.

Table S1. Reference sequences and papers for mosquito species identification

Species	GenBank	Reference*
Aedes albopictus	MN080760	Motok et al., 2019
Ae. vexans	MK402915	Ruiz-Arrondo et al., 2019
Ae. aegypti	MN299016	Lapadula et al., 2020
Ae. malayensis	MG921176	Motok et al., 2018
Armigeres subalbatus	KF406787, AY440299	Ashfaq et al., 2014; Bartholomay et al., 2004
Culex pipiens quinquefasciatus	MG712556	Lawrence et al., 2019
C. gelidus	HQ398895	Cook et al., 2010
C. vishnui	AB738195	Pham Thi et al., 2017
C. pseudovishnui	KF564723	Chan et al., 2014
C. tritaeniorhynchus	HQ398885, AB690851	Cook et al., 2010; Kuwata et al., 2012
C. pallidothorax	KY856958	Phanitchakun et al., 2017
C. fuscanus	KF407917	Ashfaqet al., 2014
C. sitiens	MN398411	Low et al., 2020
C. cinctellus	AB738110	No
C. bitaeniorhynchus	MK170093	Camp et al., 2019
C. fuscocephala	KF406799	Ashfaq et al., 2014
Mansonia uniformis	MG712564	Lawrence et al., 2019
Anopheles barbirostris	AB436010	Suwannamit et al., 2009
An. tessellatus	KF564699	Chan et al., 2014
An. aconitus	DQ000264	Junkum et al., 2005
An. vagus	MH425409	Vu et al., 2018
An. sinensis	KX779551	Feng et al., 2017
An. kochi	MK893424	Jatuwattana et al., 2020

*References

Ashfaq, M., Hebert, P. D., Mirza, J. H., Khan, A. M., Zafar, Y., Mirza, M. S., et al. (2014). Analyzing mosquito (Diptera: culicidae) diversity in Pakistan by DNA barcoding. *PLoS One* 9, e97268. doi: 10.1371/journal.pone.0097268.

Bartholomay, L. C., Cho, W. L., Rocheleau, T. A., Boyle, J. P., Beck, E. T., Fuchs, J. F., et al. (2004). Description of the transcriptomes of immune response-activated hemocytes from the mosquito vectors *Aedes aegypti* and *Armigeres subalbatus. Infect. Immun.* 72, 4114-4126.

Camp, J. V., Karuvantevida, N., Chouhna, H., Safi, E., Shah, J. N., and Nowotny, N. (2019). Mosquito biodiversity and mosquito-borne viruses in the United Arab Emirates. *Parasit. Vectors* 12, 153. doi: 10.1186/s13071-019-3417-8.

Chan, A., Chiang, L. P., Hapuarachchi, H. C., Tan, C. H., Pang, S. C., Lee, R., et al.

(2014). DNA barcoding: complementing morphological identification of mosquito species in Singapore. *Parasit. Vectors* 7, 569. doi: 10.1186/s13071-014-0569-4.

Cook, S., Lien, N. G., McAlister, E. and Harbach, R. E. (2010). Bothaella manhi, a new species of tribe Aedini (Diptera: Culicidae) from the Cuc Phuong National Park of Vietnam based on morphology and DNA sequence. *Zootaxa* 2661, 33-46.

Feng, X., Huang, L., Lin, L., Yang, M., and Ma, Y. (2017). Genetic diversity and population structure of the primary malaria vector *Anopheles sinensis* (Diptera: Culicidae) in China inferred by cox1 gene. *Parasit. Vectors* 10, 75. doi: 10.1186/s13071-017-2013-z.

Jatuwattana, W., Saeung, A., Taai, K., Srisuka, W., Thongsahuan, S., Aupalee, K., et al. (2020). Systematic studies of *Anopheles (Cellia) kochi* (Diptera: Culicidae): Morphology, cytogenetics, cross-mating experiments, molecular evidence and susceptibility level to infection with nocturnally subperiodic Brugia malayi. *Acta Trop*. 205, 105300. doi: 10.1016/j.actatropica.2019.105300.

Junkum, A., Komalamisra, N., Jitpakdi, A., Jariyapan, N., Min, G. S., Park, M. H., et al. (2005). Evidence to support two conspecific cytological races on *Anopheles aconitus* in Thailand. *J. Vector Ecol.* 30, 213-224.

Kuwata, R., Hoshino, K., Isawa, H., Tsuda, Y., Tajima, S., Sasaki, T., et al. (2012). Establishment and characterization of a cell line from the mosquito *Culex tritaeniorhynchus* (Diptera: Culicidae). *In Vitro Cell. Dev. Biol.-Animal* 48, 369-376.

Lapadula, W. J., Marcet, P. L., Taracena, M. L., Lenhart, A., and Ayub, M. J. (2020). Characterization of horizontally acquired ribotoxin encoding genes and their transcripts in *Aedes aegypti. Gene* 754, 144857. doi: 10.1016/j.gene.2020.144857.

Lawrence, A. L., Batovska, J., Webb, C. E., Lynch, S. E., Blacket, M. J., Šlapeta, J., et al. (2019). Accurate identification of Australian mosquitoes using protein profiling. *Parasitology* 146, 462-471.

Low, V. L., Wong, M. L., Liew, J. W. K., Pramasivan, S., Jeyaprakasam, N. K., and Vythilingam, I. (2020). Gender beyond male and female: Occurrence of a gynandromorph in the Japanese encephalitis vector *Culex sitiens* (Diptera: Culicidae). *Acta Trop*. 201, 105207. doi: 10.1016/j.actatropica.2019.105207.

Motoki, M. T., Miot, E. F., Rueda, L. M., Vongphayloth, K., Phommavanh, N., Lakeomany, K., et al. (2018). First record of *Aedes (Stegomyia) malayensis* Colless (Diptera: Culicidae) in the Lao PDR, based on morphological diagnosis and molecular analysis. *U.S. Army Med. Dep. J.* 1-18, 1-7.

Motoki, M. T., Fonseca, D. M., Miot, E. F., Demari-Silva, B., Thammavong, P., Chonephetsarath, S., et al. (2019). Population genetics of *Aedes albopictus* (Diptera: Culicidae) in its native range in Lao People's Democratic Republic. *Parasit. Vectors* 12, 477. doi: 10.1186/s13071-019-3740-0.

Pham Thi, K. L., Briant, L., Gavotte, L., Labbe, P., Perriat-Sanguinet, M., Cornillot, E., et al. (2017). Incidence of dengue and chikungunya viruses in mosquitoes and human patients in border provinces of Vietnam. *Parasit. Vectors* 10, 556. doi: 10.1186/s13071-017-2422-z.

Phanitchakun, T., Wilai, P., Saingamsook, J., Namgay, R., Drukpa, T., Tsuda, Y., et al.

(2017). Culex (Culiciomyia) sasai (Diptera: Culicidae), senior synonym of *Cx. spiculothorax* and a new country record for Bhutan. *Acta Trop.* 171, 194-198.

Ruiz-Arrondo, I., McMahon, B. J., Hernández-Triana, L. M., Santibañez, P., Portillo, A., and Oteo, J. A. (2019). Surveillance of mosquitoes (Diptera, Culicidae) in a northern central region of Spain: implications for the medical community. *Front. Vet. Sci.* 6, 86. doi: 10.3389/fvets. 2019.00086.

Suwannamit, S., Baimai, V., Otsuka, Y., Saeung, A., Thongsahuan, S., Tuetun, B., et al. (2009). Cytogenetic and molecular evidence for an additional new species within the taxon *Anopheles barbirostris* (Diptera: Culicidae) in Thailand. *Parasitol. Res.* 104, 905-918.

Vu, T. H., Andrianov, B. V., Gorelova, T. V. and Gordeev, M. I. (2018). Haplotypic diversity of mosquitoes of the genus Anopheles (Diptera, Culicidae) of North Vietnam. *Ecological genetics* 16, 18-25.

Table S2. CO1 nucleotide sequence identities of nine unconfirmed mosquito samples from Hainan to the most similar mosquito species in NCBI

Code	GenBank	Location	Nucleotide identity (%)	Species (GenBank)
W1	MT613992	WZS	91.21	*Armigeres subalbatus* (MK248724)
W2	MT575769	TC	96.37	*Culex gelidus* (MK724071)
W3	MT576036	TC	87.95	*Anopheles earlei* (AF425843)
W4	MT575771	LS	88.84	*Aedes sierrensis* (JF868947)
W5	MT575770	LS	92.75	*Aedes galloisi* (LC054361)
W6	MT596915	LS	92.71	*Aedes albopictus* (KY765461)
W7	MT606009	LS	90.56	*Aedes caspius* (MH559350)
W8	MT590372	QH	89.94	*Aedes lineatopennis* (AB738114)
W9	MT586701	QH	90.14	*Harnischia. sp* (KP902778)

Table S3. Haplotype distribution in *Aedes albopictus* populations

Location	HK	WC	LD	SY	LG	DZ	DF	QH	WN	LS	TC	WZS
Hap1	20	11	19	24	9	11	7	11	16	30	27	19
Hap2	2											
Hap3	2										2	
Hap4	1											
Hap5	1	3	10	9	3	1	12	2	5	13	9	4
Hap6		4								2		
Hap7		1										
Hap8		1				1				1		
Hap9			1									
Hap10			4									
Hap11			1			1					1	
Hap12				1								
Hap13				1								
Hap14				1								
Hap15				1								
Hap16				1								
Hap17						2						
Hap18						1						
Hap19						1						
Hap20						1						
Hap21						2						
Hap22							1					
Hap23							9					
Hap24								1				
Hap25								1				
Hap26											1	
Hap27											1	
Hap28											1	
Hap29											1	
Hap30											1	
Hap31											2	
Hap32												1
Hap33												1
Hap34							1					
Hap35							1				1	
Hap36							1					
Hap37							1					
Hap38	2	1										
Hap39				2								
Hap40				1								
Hap41										1	1	

Hap42										1
Hap43										1
Hap44	1									
Hap45	1									
Hap46									1	

Table S4. Analysis of molecular variance (AMOVA) of *Aedes albopictus* populations

Source of variation	d.f.	Sum of squares	Variance components	Percentage of variation
Among populations	11	18.752	0.04102Va	7.32
Within populations	340	176.5	0.51912Vb	92.68

d.f., degrees of freedom. Fst = 0.07, $P < 0.001$, Nm = 6.64.

Table S5. Haplotype distribution in *Armigeres subalbatus* populations

Location	HK	WC	SY	DZ	LS	WZS	A*	B*
Hap1	20	8	2	21	3	6		60
Hap2	3	2		3				8
Hap3		5	9	5	9	12		40
Hap4		2						2
Hap5	2	5		1	1	2	11	
Hap6				2		2		4
Hap7				1			1	
Hap8					1			1
Hap9					1			1
Hap10						1		1
Hap11	1						1	
Hap12		2						2
Hap13			3					3
Hap14			1					1
Hap15						3	3	
Hap16						3	3	

*A and B are two subgroups according to the phylogenetic tree (Fig. 3B).

Table S6. Analysis of molecular variance (AMOVA) of *Armigeres subalbatus* populations

Source of variation	d.f.	Sum of squares	Variance components	Percentage of variation
Among populations	5	30.296	0.19036Va	10.48
Within populations	136	221.214	1.62657Vb	89.52
Between subgroup	1	82.743	2.49127Va	77.06
Within subgroup	140	103.827	0.74160Vb	22.94

d.f., degrees of freedom. Population $F\text{st} = 0.10$, $P < 0.01$, Nm = 4.5. Subgroup $F\text{st} = 0.77$, $P < 0.01$, Nm = 0.15.

Table S7. Pairwise genetic differentiation (*F*st; lower triangle) and gene flow (Nm; upper triangle) between different geographical populations of *Armigeres subalbatus*

Location	HN	PT	ID
HN		1.15	3.37
PT	0.30**		0.75
ID	0.13*	0.40**	

HN, Hainan, 142 samples. PT, Pakistan, 10. ID, India, 7.

*, $P < 0.05$. **, $P < 0.01$. When *F*st is negative, Nm is not available.

Table S8. Haplotype distribution in *Culex pipiens quinquefasciatus* populations

Location	HK	LD	SY	DZ	DF	QH	WZS
Hap1	60	29	61	72	36	15	89
Hap2	18						
Hap3		8	4				
Hap4		1					
Hap5		3					
Hap6		2					
Hap7		3					
Hap8		1					
Hap9		1					
Hap10		1					
Hap11		1					
Hap12		1					
Hap13		1					
Hap14		1			1		
Hap15		1					
Hap16				1			
Hap17				2			1
Hap18				1			
Hap19				1			
Hap20				31			
Hap21				1			
Hap22				1			
Hap23				1			
Hap24				1			
Hap25				1			
Hap26						1	
Hap27					1		1
Hap28							1
Hap29							1

Table S9. Analysis of molecular variance (AMOVA) of *Culex pipiens quinquefasciatus* populations

Source of variation	d.f.	Sum of squares	Variance components	Percentage of variation
Among populations	6	16.522	0.03806Va	9.57
Within populations	450	161.747	0.35944Vb	90.43

d.f., degrees of freedom. *F*st =0.10, $P < 0.01$, Nm = 4.50.

Table S10. Pairwise genetic differentiation (*F*st; lower triangle) and gene flow (Nm; upper triangle) between different geographical populations of *Culex pipiens quinquefasciatus*

Location	HN	SB	TK	UK	CD
HN		55.36	29.18	-	109.51
SB	0.01		-	-	7.75
TK	0.02	-0.02		-	3.64
UK	-0.01	-0.06	0.00		9.33
CD	0.01	0.06	0.12	0.05	

HN, Hainan, 457 samples. SB, Serbia, 26. TK, Turkey, 11. UK, United Kingdom, 7. CD, Canada, 7. When *F*st is negative or zero, Nm is not available.

Table S11. Haplotype distribution in *Culex tritaeniorhynchus* populations

Location	DZ	QH	TC
Hap1	1		
Hap2	2	3	5
Hap3	1		
Hap4	1		
Hap5	1		
Hap6			1
Hap7			1
Hap8			1
Hap9			1
Hap10			1
Hap11			1
Hap12		1	4
Hap13			1
Hap14			1
Hap15		2	
Hap16		1	
Hap17		1	
Hap18		2	
Hap19		1	
Hap20		1	
Hap21		1	
Hap22		1	
Hap23		1	
Hap24		1	
Hap25		1	
Hap26		1	
Hap27		1	
Hap28		1	
Hap29		1	
Hap30		1	
Hap31		2	
Hap32		1	1
Hap33		1	
Hap34		1	
Hap35		1	
Hap36		1	
Hap37		2	
Hap38		2	
Hap39		1	
Hap40		1	

Hap41	1	
Hap42	1	
Hap43	1	
Hap44	1	
Hap45	1	
Hap46	1	
Hap47	2	
Hap48	1	
Hap49	1	
Hap50		2
Hap51		3
Hap52		2
Hap53		1
Hap54		1
Hap55		1
Hap56		1
Hap57		1
Hap58		1
Hap59		1
Hap60		1
Hap61		1
Hap62		1
Hap63		2

Table S12. Pairwise genetic differentiation (*F*st; lower triangle) and gene flow (Nm; upper triangle) between *Culex tritaeniorhynchus* populations on Hainan

Location	DZ	QH	TC
DZ		34.26	4.24
QH	0.01		43.21
TC	0.11	0.01	

When *F*st is negative, Nm is not available.

Table S13. Haplotype distribution in *Culex gelidus* populations

Location	HK	DZ	QH	TC
Hap1	1			3
Hap2	7	4	3	22
Hap3				2
Hap4				1
Hap5				1
Hap6	5	2	2	6
Hap7	1			4
Hap8		1	1	6
Hap9				1
Hap10				1
Hap11				1
Hap12				1
Hap13				1
Hap14	1			1
Hap15			1	

Table S14. Pairwise genetic differentiation (*F*st; lower triangle) and gene flow (Nm; upper triangle) between *Culex gelidus* populations on Hainan

Location	HK	DZ	QH	TC
HK		-	-	-
DZ	-0.10		-	-
QH	-6.00	-0.17		-
TC	-0.02	-0.04	-0.08	

When *F*st is negative, Nm is not available.

Table S15. Haplotype distribution in *Culex pallidothorax* populations

Location	WC	DZ	WZS
Hap1	3		38
Hap2			1
Hap3	5		11
Hap4		5	
Hap5		5	
Hap6	1	3	1
Hap7		4	
Hap8		1	3
Hap9		1	6
Hap10		3	
Hap11	3		3
Hap12			1
Hap13			1

Table S16. Analysis of molecular variance (AMOVA) of *Culex pallidothorax* populations

Source of variation	d.f.	Sum of squares	Variance components	Percentage of variation
Among populations	2	37.358	0.69143Va	33.06
Within populations	96	134.394	1.39994Vb	66.94

d.f., degrees of freedom. *F*st = 0.33, $P < 0.001$, Nm = 1.01.
